# Extra Supplement—The Nursing Mirror

**Published:** 1889-01-19

**Authors:** 


The Nursing Mirror.

All Contributions for this Supplement should be addressed "The Nursing Mirror," care of The Editor, 38, Old Jewry, E.C.

### En Passant.

SHORT ITEMS.—Last week's *Lady's Pictorial* contained an excellent sketch of Miss Barton, of the Royal Free Hospital.—Miss Pitt Taylor offered a prize for the best-decorated ward at the Brompton Hospital. The Jenny Lind Gallery bore the palm.—*Nursing Notes* can now be had from Mr. Vickers, 317, Strand; or from Mr. Renshaw, 356, Strand.

DISSATISFIED PROBATIONERS.—Five of the probationer nurses at Salford Union Infirmary have requested permission to depart from that establishment. The alleged reason is that they were promised thorough training in nursing, and they find this cannot possibly be had at the infirmary. Perhaps if the guardians would make a careful investigation they would find there were other reasons why the probationers are dissatisfied; also they might discover that with a little trouble the nurses could receive a very fair amount of medical training, and it would be better to grant them facilities for this than to lose them. Dr. Conry, the medical superintendent, should be asked to explain and remedy the matter.

VERSES, OR VILIFICATION?—The departure of Miss Maitland, late nurse of the Southend Victoria Hospital, has been signalled in an unbecoming way. Whatever Miss Maitland's troubles may have been, we hope she was ignorant of the placarding of posters and distribution of verses, which one of her partisans presumably indulged in. This style of complaint is undignified, to say the least of it:—

Wanted a Matron fitted to fill
The various duties, with competent skill,
Of Nurse night and day, and Servant besides,
For a House Committee which never confides
In any official, however they toil,
In a certain small Hospital on Essex soil, etc., etc.,

We are not quite sure whether it is the metre or the matter which most annoys us in this rigmarole.

DISTRICT NURSES AT GLASGOW.—The committee of the Glasgow Sick Poor and Private Nursing Association, must be much obliged to the Marquis of Lorne for the advice he has been so kindly giving them. He bids them affiliate themselves with the Queen's Institute for Nursing the Poor, of which the Princess Louise is an office-bearer. This would of course be a good thing for the Queen Victoria nurses who are seeking in vain for a local habitation and means of subsistence. Would it not be better if the Jubilee sum granted to Glasgow were given to that most excellent institution for Nursing the Sick Poor which already exists? The remark of the Marquis that the badge and designation of Queen's Nurses would be a guarantee of their efficiency, was scarcely complimentary to the thoroughly-trained nurses now working for the institution.

QUI NON PROFICIT, DEFICIT.—It is a serious thing to take a motto like this, which has recently been, so to speak, embroidered on the banner of the nursing profession. There is no doubt that good results are to be obtained in nursing, as in all else, only by a constant striving after a higher and higher ideal of skill and earnestness, and it is only right that this skill should receive public recognition from those who are competent to judge of its reality; but it is rather strange that some of those who shout their "qui non proficit" most loudly are not, strictly speaking, certificated nurses at all. Do they raise their war-cry of three years' training, registration, and the rest of it, because they feel in their own persons the want of a strict and careful training, or because they hope to hide all deficiencies under the all-embracing cloak of a nurses' register?

GROWLS AND GRUMBLINGS.—The *Daily News* has published many more letters from discontented pros., till the *Globe* has been obliged to come to the conclusion that there is no smoke without fire, and that really probationers must be badly treated. How is it that all these letters which have appeared in our contemporary deal with only one side of the question? Is it because those who are contented can afford to smile, and have no time for writing to the papers? Once start a fit of grumbling, and it is a most infectious complaint: a probationer began by quarrelling with her bread-and-butter, but now not only is the nursing staff, but the medical staff and the whole hospital coming in for condemnation at the hands of various correspondents. We do not sympathise with this spirit in the least, and advise our readers to note the letter from a sister which we published last week, which deals with this subject in the way it deserves.

NURSE OR MOTHER?—In his speech at the annual meeting of the Glasgow Royal Infirmary, Professor Dunlop told a sad little story, which, however, has a bright side for us nurses. Six weeks ago a boy of twelve was admitted to the infirmary, and as it soon became evident that the disease from which he was suffering would prove fatal, his mother was sent for. The day after the mother's visit the surgeon spoke to the child about her and her love, and did not at first understand what emotion it was that caused his tears to flow so rapidly; but the nurse explained that when the mother came she was intoxicated, that her presence brought little comfort to the dying boy. On hearing this, the doctor led the nurse to the bed, placed the child's hand in hers, and told him that here was someone who would be a mother to him. The little fellow accepted the substitute gladly. He never spoke of his mother again, but entered the dark valley content and happy, holding his nurse's hand. After all, it is no mean privilege that comes to a nurse sometimes, of showing something of mother-love to those little ones whom circumstance has taught to regard the bond between parent and child with more of fear than tenderness.

THE ORDER OF ST. JOHN.—Strangely enough, the nursing branch of the Order of St. John of Jerusalem is scarcely known to the public at all, though the ambulance department is the talk of every household. We quote the following from the last report of the nursing branch to show how rapidly its work is increasing: "In presenting a report for the past year the managing committee are again able to speak most satisfactorily of the work done by St. John's House Nursing Association. The centres of work among the poor are as follows: Worcester, Kidderminster, Bromsgrove, Droitwich, Malvern, Ely, and St. Mary Church, Torquay. But the association has gone further afield than England. At the request of the Order of St. John, a nurse was sent last August to Jerusalem, and is now working in the Ophthalmic Hospital of that city. During the past year one probationer has undergone the usual course of hospital training, and is now working satisfactorily in Worcester. One ambulance pupil has also passed two months' training in St. John's House. There are now ten nurses on the staff, and in addition four supernumeraries. The new year brings with it a new work. Through the generosity of Mrs. Perrins, of Davenham Bank, a convalescent home for twelve patients will shortly be opened at Malvern, and by the request of the foundress two nurses from St. John's House will take charge of the home, and will at the same time continue their district nursing. Miss Topping, the superintendent of the Worcester Branch, carries out her duties most admirably, and at the annual meeting, held at Worcester on January 7th, she came in for special praise from the Bishop of Ely and Sir E. A. H. Lechmere.

### Original Articles.

### A MONTH IN HOSPITAL.

By One Who Has Tried It.

Like most healthy Englishmen, I had an instinctive dread of a hospital, which, to my mind, conjured up scenes of sickness and suffering, ghastly sights and sounds, and all the attendant effects on the nerves which are generally connected with that dreaded name. However, a serious accident, and subsequent complication of rheumatism and loss of power, rendered it necessary for me to "lie up" for a few weeks, under medical care, and I was persuaded to go to London and enter myself as a paying patient at a well-known hospital. I shall never regret having done so, and strongly advise anyone really suffering bodily ills to "go and do likewise."

On arriving, I was shown into a waiting-room, and supplied with a printed form, on which I wrote my name and address, and the address of my nearest relative or friend in case of my decease. I then went to the steward's office, where I paid my fees for a week, which amounted to £2 16s.—*i.e*, 8s. per day. (This includes board and lodging and medical attendance.) I was then taken upstairs in the "lift" to Ward No. 2, and as I entered the long, comfortably-warmed room, with its double windows and doors shutting out the noisy hum of humanity without, I could easily imagine I was in any other place than busy London.

Ward II. is a long, lofty room, warmed with hot-air stoves and two open fires in the centre. Down each side are curtained partitions of striped holland, forming little compartments for the patients, which are indicated by letters A, B, etc., painted on the wall over each. These compartments contain a bed, chest of drawers, washstand, looking-glass, and two chairs. The curtains may be drawn or left open at pleasure, so that strict privacy is as easy as in a separate room, for no one but a nurse or doctor enters a "closed compartment."

On my arrival a nurse, who was sitting near one of the centre tables, rose and came towards me, and when I explained I was a patient she led the way to a compartment lettered K, and asked if I would go to bed at once. Being tired and in pain from my railway journey (I had come from Yorkshire that morning) I was only too glad to get into bed, and my nurse quickly and skilfully assisted me to undress. In a few moments I was quite comfortable, with a hot bottle at my feet, whilst nurse quietly unstrapped my portmanteau and arranged my belongings in the drawers at the bedside. A hot basin of good beef-tea was a welcome meal, and I rested comfortably for the remainer of the afternoon.

Late in the day the doctor paid his round of visits, and had a chat with me, and finding there was no immediate attention necessary, and that mine was a somewhat complicated case: he advised me to have a specialist to meet him in consultation, to whom I should have to pay a fee of £2 2s. To this I gladly agreed, and the next morning the two doctors made a careful examination of me all over, and retired for consultation. On returning they informed that my treatment was to be hot baths and medical rubbing, with suitable diet, and perfect rest for a month. The medical rubbing or "massage" is done by a professional man not attached to the hospital, and to paying patients his fee is 5s. an hour. I had an hour every other day, as it was as much as I could stand in my weak state.

The day after I entered the hospital I began to make acquaintances; those of my fellow patients who were convalescent, paid me visits to inquire how I was, and offer books, papers, fruit and flowers, with which they were liberally supplied by their friends. I had already been presented to the night nurse, who looked in several times during my first (and only) wakeful night, and brought me milk and soda. At a quarter to seven a.m. the ward-maid brings round breakfast, which consists of tea, coffee or milk, and bacon, eggs, fish, toast, and bread-and-butter, with jam or marmalade *ad lib.* Then at half-past seven comes the newspaper boy, followed by the barber; at eight the ward-maids sprinkle tea leaves on the polished oak floor, and carefully sweep the ward from end to end, whilst the convalescent patients retire either to the reading-room, or closed corridor, to smoke.

About nine o'clock the doctor pays his morning round of visits; and at eleven, lunch of sandwiches or beef-tea is served. Between eleven and one is operation time. The patient to be operated on is taken into a large heated room at the end of the ward, where the doctors, matron, and nurse assemble. Not a sound is heard; as under the influence of ether, the most difficult and miraculous operations are performed, of which the patient knows nothing; and the calm face of the sufferer, as the little white bed is wheeled along the ward to its compartment, gives no indication of the effects of the dreaded knife. At one o'clock dinner is served in the dining-room, for those who are sufficiently well to get up; and in bed for the rest. The dining-room fare is generally beef or mutton—boiled and roast, a pudding, cheese, and vegetables. To those in bed a lighter fare of fish, boiled or roast chicken, and milk pudding; stout, port wine, or whisky, at doctor's discretion.

From one to three is a quiet time to get a nap, as our visitors arrive at three, and may remain until five every day. These two hours are looked forward to by each patient who has friends in London, and generally the thoughtful care of some lady relatives or friends infuses an atmosphere of brightness to the little compartment by a gift of fresh flowers, or a basket of fruit. At five o'clock, tea is served; eggs, jam, and bread-and-butter for those who care for it. Then comes the general adjournment to the smoking-room, where the incidents of the day or pending operations are discussed, and the timorous ones are cheered by the convalescent ones who have already "gone through it," by being assured they "will not feel it," and "will soon get over it," etc.

At half-past seven the bath-man came to "tub" me, and I was carefully "boiled" for twenty minutes, then lifted out, dried, packed up in a blanket, and lifted into bed; there I lay still for half an-hour to cool. At half-past eight I had my supper of sandwiches (or beef tea) and a tablespoonful of whisky with soda water.

The day-nurses come on duty at eight a.m., and each one has two hours "off" during the day. They remain on duty until ten p.m., when the night-nurses relieve them. Each nurse in the Home attends to six beds, so they have more time for each patient than in the general hospital, when they have more than double that number under their care. After the first day or two, time passes away very pleasantly in the Home, and I never heard but one opinion expressed by patients going out, and that was they were "sorry to leave." Before my month had elapsed, I began rapidly to recover, and three days before my appointed time I was considered sufficiently well to go home, and though I naturally felt delighted once more to feel the full use of my limbs, it was not without sincere regret that I went round to each bed and shook hands with my comrades in adversity. Lastly I bade farewell to my kind nurse, and promising to call whenever I was in London, I left the Home and went out into the world.

I may say, in conclusion, that nothing gives the hospital nurses more pleasure that a visit from an ex-patient, as it proves to them more than any other expression of thankfulness that you appreciate their ministrations.

### The Entertainment at St. Bartholomew's.

The entertainment to the resident staff at Bart's this year consisted of a concert and a dramatic performance, sustained chiefly by workers in the hospital. The stage, which was erected at the end of the board-room, presented a pretty aspect when the long line of nurses in their trim uniforms, with the darker line of members of the Hospital Musical Society behind them, struck up the appropriate opening glee, Pearsall's "Come, let us be Merry." The rendering of this and the other glees—Schumann's familiar "Wanderer's Song;" a quaint little song by Farmer, "To Take the Air;" and Pinsuti's musicianly "Song to Pan"—proved that the hospital may well be congratulated on its musical talent, though the singing of Mr. and Mrs. Robert Price must have given special pleasure to an audience that has not much time to go out to concerts. Both Mr. and Mrs. Price are fortunate in possessing good voices and a cultivated style, and both in their solos ("Last Night," given by Mrs. Price, and "Like to Like," by her husband) and in Lucantoni's duet, "A Night in Venice," delighted their listeners. Coleman's comedy, *The Heir-at-Law*, which formed the second part of the performance, was creditably played by members of the Hospital Amateur Dramatic Club. The most finished performance was that of Mr. A. Johnson as Daniel Dowlas, in which he showed an ease and vigour not common among amateurs, though the humours of Doctor Pangloss, and Kenrick, the Irish servant, were duly accentuated by Mr. J. Valerie and Mr. L. C. Thorne-Thorne. Mr. H. L. Brownlow, as Zekiel Homespun, was sympathetic and intelligent: but the part is a very difficult one, both in its humour and its pathos. The hero, full of orthodox sentiments, has always rather an ungrateful part, and it may be forgiven Mr. G. J. Roughton if he was rather stiff as Henry Morland; and his friend Stedfast, played by Mr. J. P. Graham, is overweighted by moralising speeches of a similar kind. The female parts of the comedy are comparatively unimportant, but Miss Barnes showed considerable sense of comedy as Deborah Dowlas; and Misses F. and A. Newton made the most of the *rôles* of Caroline Dormer and Cicely Homespun. To an outsider the audience was not the least interesting part of the performance. It is not often that one sees such a becoming uniformity of garb among ladies, and the visitors, in evening dress, looked quite "out of it" against the neat caps and simple gowns of the nurses, and certainly could not compete with their aspect of sincere and hearty enjoyment, which proved conclusively that the entertainment was a great success.

### ARE NURSES SHORT-LIVED?

It has been stated with much decision, and more than once of late, that nurses are short-lived, whereas our experience shows the contrary, a fact which had a material influence upon the rates fixed by the National Pension Fund. Some interesting evidence on the point has been brought to our notice during the present week. Twelve trained nurses who have been pensioned by a society, afford excellent proof of the robust constitutions which trained nurses of long-standing usually possess. The average age of these pensioners is 76, the oldest being 86 years, and the youngest 67. The ages of the others are as follows: one is 82, one 78, two are 77, three are 75, one is 74, and two are 70. We always like to hear both sides of the question, and should therefore be glad to know what those who contend that a nurse is worn out at 50, have to remark on the foregoing facts.

### NURSE MATRON IN FIJI.

Mr. Burdett desires to thank the lady superintendents, matrons, and others who have been good enough to communicate with him in respect to this appointment. The applications are too numerous to render it possible to acknowledge each separately, and Mr. Burdett hopes that his correspondents will kindly accept this intimation in lieu of a letter. The appointment will be made immediately, and no further applications can be entertained.

### INTERVIEWING A MATRON.

The *Star* reporter has been calling on Miss Hicks, at Great Ormond Street, and publishing his interview. He discovered that in 1885 Miss Hicks was selected as superintendent of the Princess of Wales's branch of the Red Cross Society to go to Egypt. She and seven more went out as far as Suez, and then Miss Hicks took one and commenced her journey towards the country of the Mahdi. She went up the Nile. The others stayed at Suakin. From March till the end of July Miss Hicks and her companion were under canvas. The heat was killing, although they lived in double tents. Then the soldiers made mud huts and laid palm leaves over them. The following conversation is also given by this American style of journalist:

"The soldiers were perfect in their chivalry and patience," said the matron. "A vast number of deaths occurred. Water was very scarce—no one had more than a gallon for drinking and washing purposes. It had to be boiled directly it was drawn from the well, or in a short time tadpoles would swarm in the bucket. Fruit was our principal diet."

"Of course, Miss Hicks, you had some assistance?"—"Yes; the orderlies. But they died off very rapidly. No one has any idea of the sick soldier. When a man cuts his finger at home he makes a terrible fuss; but out there men bore the keen sufferings from diseases and wounds without a murmur."

### THE BRITISH NURSES' ASSOCIATION.

A general meeting of the council of the British Nurses' Association was held yesterday in the Medical Society's Rooms, Cavendish-square, under the presidency of Mr. Savory. The annual report of the committee, which was read by Dr. Bedford Fenwick, stated that the Association had proved to be in every way most successful in its initiation and in its progress during the eleven months of its existence. Immediately after its inauguration 100 members were enrolled, and ever since the membership had gone on steadily increasing, till now they reached a total of 2,203. Permission had also been granted for the formation of branches in the Australasian colonies and South Africa. The receipts had amounted to £736, against an expenditure of £216. The committee felt the great importance of arousing public opinion to the necessity that existed for the registration of trained nurses, and they proposed to take steps to accomplish that end. The future work of the Association would include the foundation of a benevolent fund to assist such members as might be in need of temporary pecuniary assistance; the foundation of a medal of merit for nurses; and the establishment of convalescent and holiday homes in the country or at the seaside. The committee concluded by acknowledging the assistance the Association had derived from the advice and support of her Royal Highness Princess Christian. On the motion of Dr. Schofield, seconded by Miss Mollett, the report was adopted.

[It will be noticed that the total number of members of all grades is stated to be 2,203, but no details as to the number of nurses who have joined are supplied. Assuming that the whole of the £736 received was derived from members' subscriptions, it will be seen that the doctors, matrons, and other persons who pay half-a-guinea a-year, must number something over 1,150, and the nurses, who subscribe 2s. 6d. each, something under 1,050.—Ed. *T. H.*]

### Everybody's Opinion.

*[Correspondence on all subjects is invited. No communications can be entertained if the name and address of the correspondent is not given, or unless one side of the paper only be written on.]*

### THE BRITISH NURSES' ASSOCIATION.

A Member of the B.N.A. writes: In case you had no reporter present at our general meeting last night, I send you word that we had a fair attendance of members, and Dr. Fenwick gave a good report of progress. He said that the Association meant to start in time a benevolent fund for nurses, a convalescent home, and a medal. I don't like the idea of a medal at all, but I suppose it will appeal to some people. Also I hear that Miss Wood spoke at a meeting at Shrewsbury lately, and told the nurses there that they committed themselves to nothing by joining the Association; and also that the Association was quite ready to take up, and, if possible, obtain a charter for the registration of midwives. You will see, then, we have our work cut out.

### REST AND EXERCISE.

Nurse Jane writes: "I have read with much regret M. B.'s letter, as from my experience it conveys such a wrong impression of nursing institutions. I have belonged to one for some time, and have always been treated with kindness and consideration, as have all the nurses, rest and exercise being especially cared for. We have seven hours for sleep and one for exercise, and, when possible, a day or two's rest in the Home after a hard case. If any nurse is ill she is kindly and tenderly nursed, and if a holiday is necessary a suitable place is found, her salary going on just the same. I know of many institutions where it is the same. It is not true that nurses are sent away when thirty-five; many here are more than that."

### PRIVATE NURSES AND NURSING INSTITUTIONS.

A Private Nurse requests to be allowed to suggest to the readers of The Hospital that all private nurses combine for their own good in petitioning their different committees to grant them all fees over £1 1s. per week paid for their services. If the nurse derives no benefit from the extra fee, charged because she is overtaxed, why need it be charged at all? Matrons will answer, "Because at the end of the case, the nurse requires a change." Yes, but unless she is thoroughly ill, the only change she gets is a fresh case. For instance, I had been with a very trying mental patient for four months (the fee for which was £2 2s. per week), and on the same day that I returned, sadly in need of a rest, I was, in less than forty minutes, packed off to another case. Now, if the extra £1 1s. had been allowed to me, who had, in the consideration of my employers, justly earned it, how nicely it would have come in for the Pension Fund. Instead of this, I handed over a cheque for nearly £34, and received in return the ridiculous sum of £5 for my quarter's salary. Is not this truly "feathering another person's nest"? The institution does not hand its profits over to charity; it is private, and belongs to two "hospital sisters."

### CHILDREN AND NURSES.

"As an example of the way in which the children regard their nurses, I will tell two little stories that I heard during my visit. A very small boy had been there for some time, and when he was taken home cured, he left his brother behind him there, still an invalid. But he could not reconcile himself to his old home. 'Mother,' he said, 'I wish you would take me back to where the angels live.' His mother not understanding at first what he meant, he added, 'to the place where Bob still is, mother.' The other story is also of a little fellow who fell in love with his nurses while in the hospital. Some weeks after he had left he returned to pay them a visit. He had one fist, more chubby than it was when he was an inmate, screwed up tightly. He held it out to the nurse, who, on opening it, found the sum of 2s. 11d., which the boy had collected among his friends, and brought for the benefit of the nurses and their hospital."—*Birmingham Daily Mail.*

### Appointments.

[It is requested that successful candidates will send a copy of their applications and testimonials, with date of election, to The Editor, The Lodge, Porchester Square, W.]

Llanelly Hospital.—Miss Rachel Edwards, of the Queen's Hospital, Birmingham, has been appointed matron and head nurse at Llanelly. Miss Edwards trained at St. Bartholomew's some years ago, and has had considerable experience in the various branches of nursing.

St. Helier's Infirmary.—Miss Browne, late charge-nure, at the Devon and Exeter Hospital, has been appointed matron and nurse of the infirmary attached to the St. Helier's Dispensary. Miss Browne trained at University College Hospitals which she left to go to St. Thomas's for further experience.

### Vacancies.

The following vacancies are announced :—

**Matrons.**

Bristol Royal Infirmary; £100. Bristol Eye Hospital; £40. Cromer Cottage Hospital (Nurse).

**Lady Superintendent.**

Northampton General Infirmary; £60.

**Night Sister.**

National Hospital, Queen Square, £30.

**Nurses.**

Nursing Institution, 96, 97, 98, Wimpole Street, London, W., Mr. Wilson has several vacancies for thoroughly-trained Nurses.

Llanelly Hospital; £20.

Bedford Nursing Institute; two.

Sheffield Union; £20.

Royal Albert Hospital, Devonport; several; £25.

Southport Nurses' Home.

St. Helier's Institute; £25.

Brockley Nursing Home.

Victoria Home, Bournemouth.

Rivers Street Home, Bath; £25.

Royal Alexandra Hospital for Sick Children, Brighton; £20.

Croydon General Hospital; £22.

Swansea and South Wales Nursing Institute.

Llandudno Cottage Hospital.

Southport Infirmary; £20.

Nottingham Nursing Institute; £25.

County Institution, Worcester.

Swansea Hospital; £22.

City of London Hospital; £24.

Bridgnorth Union; £25.

Sheffield Infirmary (night); £20.

County Hospital, Guildford; £16.

Nursing Institute, 5, Henrietta Street; several.

Blackheath Institute; £25.

Truro Infirmary; £25.

Nurses Home, Norwich.

Newark Hospital; £25.

Newlands, Ryde, I.O.W.; Two.

Workhouse Infirmary Nursing Association, Adam Street, Adelphi; Several.

Newport and County Infirmary, Mon.; £25.

County of Cornwall; Trained.

Nurses' Home, Truro; £25.

**District Nurses.**

Nursing Institution, Hitchin.

Bedford Nursing Instute.

Glasgow Sick Poor Association.

Bicester.

Madeley, Staffordshire.

Ryde, I.O.W.

**Probationers.**

Ellison Place Home, Newcastle-on-Tyne.

Tewkesbury Hospital.

Bolton Infirmary,

Workhouse Infirmary Nursing Association, Adam Street, Adelphi; Several.

### Notes and Queries.

To Correspondents.—1. Questions may be written on post-cards. 2. Advertisements in disguise are inadmissible. 3. In answering a query please quote the number. 4. If a private answer is desired, a stamped addressed envelope must be forwarded.

**Queries.**

(100) *Home Wanted.*—Can anyone tell me where there is a home which would take an aged and destitute architect over 80 years old?

(101) *Nursing in North of France.*—A Birmingham Hospital Nurse wishes to know the names of a few hospitals in the North of France; also if English nurses are taken in them.

**Answers.**

(93) *Home at Harrogate.*—There is a Convalescent Home being built at Harrogate, but it is not yet finished.

(97) *Home Wanted.—Nurse J.* is informed that Miss Smethurst has a home for invalids, full particulars of which may be had on application to Springfield, Beaufort Avenue, Brooklands, Cheshire. Terms from three guineas per week. An address has been sent *Nurse J.* by post.

(99) *Probationer.* — *Brighton* had better apply personally to the matron of the nearest hospital. All good London hospitals have their vacancies for probationers filled months beforehand. The age desirable to commence nursing is 25, but younger candidates of 21 are taken at provincial and children's hospitals. Wages usually commence at £10.

*The Hospital Annual.*—All who are looking for the Annual may expect to receive it early in February. The date will be duly announced.

